# Study of New Nitrogen-Fireable Copper-Nickel Thick Film Paste Formulation Compatible with Thick Printed Copper

**DOI:** 10.3390/ma15041372

**Published:** 2022-02-12

**Authors:** Jiri Hlina, Jan Reboun, Marek Simonovsky, Tomas Syrovy, Martin Janda, Ales Hamacek

**Affiliations:** 1 Department of Materials and Technology, Faculty of Electrical Engineering, University of West Bohemia, Univerzitni 8, 301 00 Pilsen, Czech Republic; jreboun@fel.zcu.cz (J.R.); jandam@fel.zcu.cz (M.J.); hamacek@fel.zcu.cz (A.H.); 2 Elceram a.s., Okruzni 1144, 500 03 Hradec Kralove, Czech Republic; simonovsky@elceram.cz; 3 Department of Graphic Arts and Photophysics, Faculty of Chemical Technology, University of Pardubice, Doubravice 41, 533 53 Pardubice, Czech Republic; tomas.syrovy@upce.cz

**Keywords:** thick-film, resistor, copper, nickel, resistive paste, electrical properties, diffusion

## Abstract

This paper is focused on a new copper-nickel thick film resistive paste which was designed and experimentally developed for the realization of low-ohmic power resistors. This copper-nickel paste has been designed for use in combination with thick printed copper conductors and in comparison with conventional ruthenium-based thick film resistor pastes allows firing in a nitrogen protective atmosphere. The copper-nickel paste was prepared from copper and nickel microparticles, glass binder particles and a combination of organic solvents optimized for its firing in a nitrogen atmosphere. This paper covers a detailed description of copper-nickel paste composition and its thermal properties verified by simultaneous thermal analysis, a description of the morphology of dried and fired copper-nickel films, as well as the electrical parameters of the final printed resistors. It has been proven by electron microscopy with element distribution analysis that copper and nickel microparticles diffused together during firing and created homogenous copper-nickel alloy film. This film shows a low temperature coefficient of resistance ±45 × 0^−6^ K^−1^ and low sheet resistance value 45 mΩ/square. It was verified that formulated copper-nickel paste is nitrogen-fireable and well-compatible with thick printed copper pastes. This combination allows the realization of power substrates with directly integrated low-ohmic resistors.

## 1. Introduction

Resistors made by well-known thick film technology have been used in various applications in electronics for decades [1]. Thick film technology is the most used and the most effective technology for hybrid electronic circuits, which covers a wide range of applications [2,3]. It is additive technology for forming conductive, resistive, or dielectric films generally on ceramic substrates [4]. These films are sequentially screen printed and after that dried and fired in the temperature range from 500 °C to 1000 °C [5].

Thick film resistors can be used in the form of discrete components or in the form of directly integrated components on electronic substrates. These resistors in principle consist of a ceramic substrate with two printed conductive terminals and printed resistive film which is located between these terminals. Standard resistive pastes for the printing of resistors are based on ruthenium, iridium, or rhodium oxides [6,7]. These pastes are usually compatible with silver or gold thick film conductors which are used for terminations [5]. The material of terminals can affect resistor properties such as its nominal resistance value due to contact resistance between terminals and resistive film or its temperature coefficient of resistance (TCR) [8]. The chemical interactions between resistive and conductive films can change the microstructure of the resistive film in the interface area and the result is higher resistance in this region [8,9]. Another issue can be the diffusion of conductive materials, especially silver, to resistive film [10,11]. Resistive and conductive pastes used for thick film resistors are determined for firing in belt furnaces under oxidative atmosphere usually at a dwell temperature of 850 °C [12,13].

Thick film technology can be also used for power hybrid integrated circuits manufacturing [14]. Hybrid integrated circuits combine ceramic substrates with printed structures and mounted passive and active discrete electronic components [12,15]. Special copper pastes are used for printing conductive patterns on ceramics (usually Al_2_O_3_ and AlN) in this case. These substrates are called thick printed copper (TPC) substrates [16,17]. TPC substrates represent an alternative solution to conventional technologies for power substrates manufacturing such as direct bonded copper (DBC) and active metal brazing (AMB) and have several benefits compared to these conventional technologies which are based on full-area copper plating and etching of required copper patterns [18,19,20]. The main benefits are the high resolution of copper patterns, a selective increase of copper film thickness up to 300 µm, high endurance to rapid temperature changes (temperature shocks), a simple realization of multilayer structures, and direct integration of passive components (based on the principle of thick film technology), which can improve the reliability of the whole hybrid integrated circuits [17,21]. However, the firing of copper films in a protective nitrogen atmosphere is necessary due to the rapid oxidation of copper in the oxidative atmosphere [22]. This fact complicates the direct integration (printing) of passive components such as resistors because standard and commercially available resistive thick film pastes are intended only for firing in an oxidative atmosphere.

Using standard resistive thick film paste in combination with copper (TPC) thick films was verified in previous research [23] where the following parameters were achieved: sheet resistance ~195 Ω/square and TCR ±120 × 10^−6^ K^−1^. A combination of firings in the oxidative and nitrogen atmosphere was necessary in this case. A combination of firings can be problematic, especially in the realization of more complex interconnections containing multilayer structures that require multiple firings in a nitrogen atmosphere. Re-firing of the resistive film in a nitrogen atmosphere during the firing of copper terminals also caused increasing of contact resistance between resistive film and printed copper terminals. Therefore, the realization of low-ohmic resistors that are predominantly used in power electronics as a shunt or current sensing resistors is difficult.

The new approach to this issue could be in the usage of thick film resistive paste or ink based on copper and nickel particles which allow firing in a nitrogen atmosphere. The alloy consists of copper and nickel in the ratio of 55:45 wt. % (so-called constantan) shows unique properties for the realization of a low-ohmic resistor. Constantan has very low TCR (±40 × 10^−6^ K^−1^) [24] and electrical resistivity 4.9·10^−7^ Ω×m. Copper-nickel nanoparticles inks are experimentally available but using this ink requires different deposition techniques, such as Ink-Jet or Aerosol Jet [25,26]. The application of copper-nickel nanoparticle ink was also verified in previous research. Resistors with the resistive film deposited by copper-nickel ink show low sheet resistance ~1 Ω/square and sufficient TCR ±100 × 10^−6^ K^−1^ [27]. However, ink formulation does not contain any adhesive components. Therefore, printed films show low adhesion on ceramic substrates and achieving higher printed film thickness is difficult. The deposition of the copper-nickel film by Aerosol Jet is a relatively slow process and difficult to transfer to industrial production. Due to the above-mentioned issues, the use of copper-nickel thick film paste and its deposition by screen-printing is preferable for mass production. However, these pastes are not commercially available and have not been described in detail in the literature yet. Thick film pastes based on nickel particles were found in the literature but mentioned pastes are designed mainly for the realization of electrodes in multilayer capacitors [28,29,30]. Copper-nickel pastes were also found in [31] but preparation involves the application of nickel-plated core-shell particles for low-temperature curing, where nickel was used to reduce copper oxidation. Therefore, this paper is focused on the preparation and detailed characterization of copper-nickel thick film resistive paste for power electronics applications.

## 2. Materials and Methods

Copper-nickel thick film paste was prepared from the mixture of Cu and Ni particles (Sigma-Aldrich, particle size < 1 µm) and glass frit particles (Heraeus, Hanau, Germany, GPA2014-013). The paste formulation was prepared by homogenization of 51.5 g of Cu particles, 42.3 g of Ni particles, and 4.2 g of glass frit paste (Heraeus, GPA2014-013) in a mixture of solvents (17.0 g of dipropylene glycol monomethyl ether and 12.9 g of terpineol) using an overhead stirrer (Heidolph Hei-TORQUE Precision 100, radial-flow impeller, Schwabach, Germany). Then, 31.5 g of the polymer solution of ethyl cellulose (Sigma-Aldrich, St. Louis, MO, USA, Ethoxyl content, 48%) was added. The final paste composition was mixed by an overhead stirrer at 500 RPM for 1 h and finally homogenized using a three-roll mill. The final viscosity of the paste was 16.3 Pa·s (Brookfield DV-I+, 100 RPM).

Thermal properties of prepared copper-nickel paste were verified by simultaneous thermal analysis (STA) using TA Instruments SDT Q600 (heat rate 10 °C/min, a nitrogen atmosphere with purity 5.0) which combines thermogravimetric analysis (TGA) and differential scanning calorimetry (DSC). Based on the results of STA analysis, the firing profile of copper-nickel paste was optimized.

**Figure 1:**
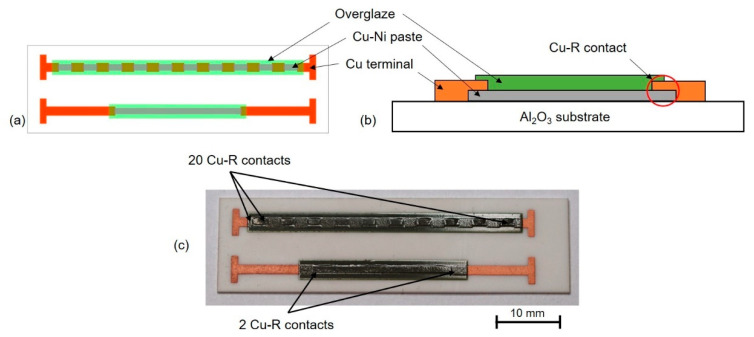
(**a**) IRP design, (**b**) schematic section of the printed resistor and (**c**) realized IRP specimen.

An intermittent resistor pattern (IRP) was used for verification of the functionality of copper-nickel paste and also for verification of contact resistance between copper films (terminals) and copper-nickel resistive films (Figure 1). The IRP is described in detail in [23]. This pattern consists of two resistive structures with the same lengths (1 mm width and 20 mm length which corresponds to 20 squares) and with a different number of contacts between copper terminals and copper-nickel resistive film (2 Cu-R contacts or 20 Cu-R contacts).

The copper-nickel paste was screen-printed on 96% Al_2_O_3_ substrates with a thickness of 0.6 mm, dried at 125 °C for 10 min and then fired in the batch furnace with a nitrogen atmosphere at a temperature of 960 °C (10 min dwell time on peak temperature, total firing time 4 h). The next step was the screen-printing of copper terminals with copper paste Heraeus C7403 that was also dried at 125 °C for 10 min and fired in the batch furnace with a nitrogen atmosphere at a temperature of 960 °C (10 min dwell time on peak temperature, total firing time 4 h). Since the firing conditions are the same for both copper-nickel and copper films, the manufacturing process is simplified and also cofiring is theoretically possible. Fired copper-nickel films were covered with nitrogen fireable overglaze Heraeus IP7098 which protects copper-nickel films against environmental effects. Overglaze paste was also screen-printed, dried at 125 °C for 10 min, and fired in the batch furnace with a nitrogen atmosphere at a temperature of 680 °C (5 min dwell time on peak temperature, total firing time 3 h). Copper, copper-nickel, and overglaze pastes were printed by semi-automatic screen-printer Ekra XH STS under the same printing conditions (stainless steel screen, mesh: 200 threads/inch, emulsion over mesh thickness: 50 µm, snap-off: 2 mm, trailing edge squeegee, squeegee speed: 25–30 mm·s^−1^, squeegee pressure: 30–40 N).

The structure of fired copper-nickel films was observed by SEM microscope Phenom ProX and EDS analysis was performed.

The temperature dependence of resistance of realized IRP specimens was measured in the temperature range from 0 °C to 100 °C with the step of 10 °C in the thermostatic bath Lauda PJL 12 with silicon oil and TCR was calculated from measured values (by the standard IEC 60751:2008) according to following formula

**Figure 2:**
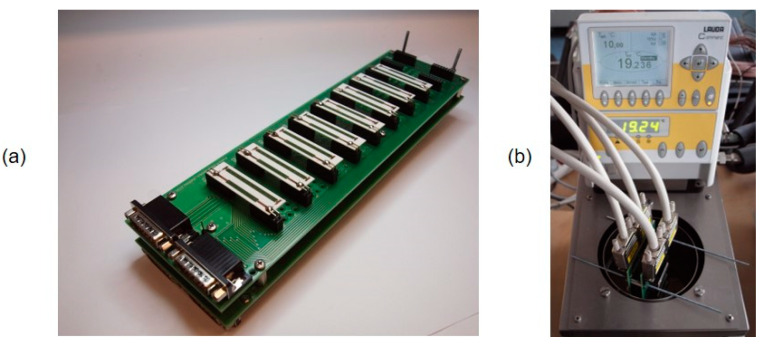
(**a**) Measurement tool for multiple measurement of resistors and (**b**) measurement tool in the thermostatic bath.

**Figure 3:**
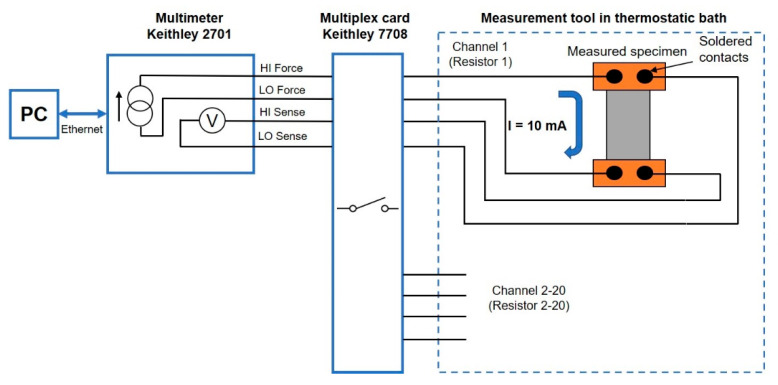
Block diagram of four-wire measurement.

TCR = (1/R_ref_) × (R−R_ref_)/(T−T_ref_) (K^−1^) (1)

where R is resistance value, R_ref_ is resistance value at 0 °C and T_ref_ is the reference temperature (0 °C). Temperature dependence of resistance was also measured after accelerated aging by dry heat test (155 °C for 1000 h according to the standard IEC 60068-2-2). Resistance of specimens was measured by the four-wire resistance measurement method which eliminates the influence of transient resistances and resistance of measurement wires. A digital multimeter Keithley 2701 with multiplex card Keithley 7708 in combination with a designed tool for the multiple measurement of resistors in the thermostatic bath was used (Figure 2). The block diagram of measurement is described in Figure 3.

## 3. Results and Discussion

**Figure 4:**
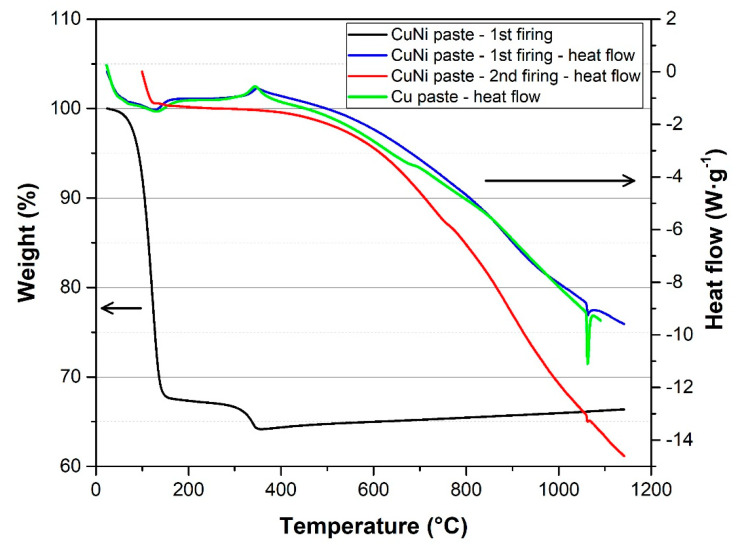
STA analysis of copper-nickel thick film paste (nitrogen atmosphere, 5.0 nitrogen purity).

Results of STA analysis of copper-nickel paste are shown in Figure 4. STA analysis proved that copper-nickel paste contains 66 wt. % of solids after firing and all organic solvents evaporate from paste formulation at temperatures up to 350 °C.

The heat flow curve of copper-nickel paste (first firing) contains several peaks which represent endothermic and exothermic reactions. The endothermic reaction at temperatures from 80 °C to 160 °C corresponds with the removal (evaporation) of solvents dipropylene glycol monomethyl ether and terpineol. The exothermic reaction at a temperature around 330 °C corresponds with the thermal decomposition of the polymer solution of ethyl cellulose [32]. The gradual diffusion of copper and nickel particles and formation of copper-nickel alloy occurs from the temperature of 400 to 500 °C. The diffusion of copper and nickel particles occurs at lower temperatures due to micrometer particle size compared to bulk copper and nickel. The endothermic reaction at a temperature of 1085 °C represents the melting point of copper. It is obvious that most copper and nickel particles formed a copper-nickel alloy because the specific enthalpy (energy of endothermic reaction) of the copper-nickel paste at this temperature (specific enthalpy 17.91 J·g^−1^) is significantly lower as compared to pure copper thick film paste (specific enthalpy 62.75 J·g^−1^), which is used for the printing of copper terminals and conductive paths. If copper-nickel alloy was not formed when this temperature was reached, the specific enthalpy in the case of copper-nickel paste would correspond to about half of the specific enthalpy of the copper paste. This fact indicates that copper and nickel particles have been diffused together. However, the specific enthalpy of the endothermic reaction at 1085 °C (melting of remaining copper particles) indicates that a certain amount of non-diffused copper particles remains in the copper-nickel film after the first firing. This amount of non-diffused copper particles is significantly lower after the second firing (the specific enthalpy of endothermic reaction at 1085 °C is 5.44 J·g^−1^). It is obvious that repetitive firing improves the properties of the final copper-nickel film (diffusion of copper and nickel particles, homogeneity and grain size). In order to ensure perfect diffusion, it is advantageous to print and fire the copper-nickel resistive paste first followed by printing and firing of copper paste (terminals). This ensures that the copper-nickel paste will pass two firings and copper and nickel will be well-diffused together.

**Figure 5:**
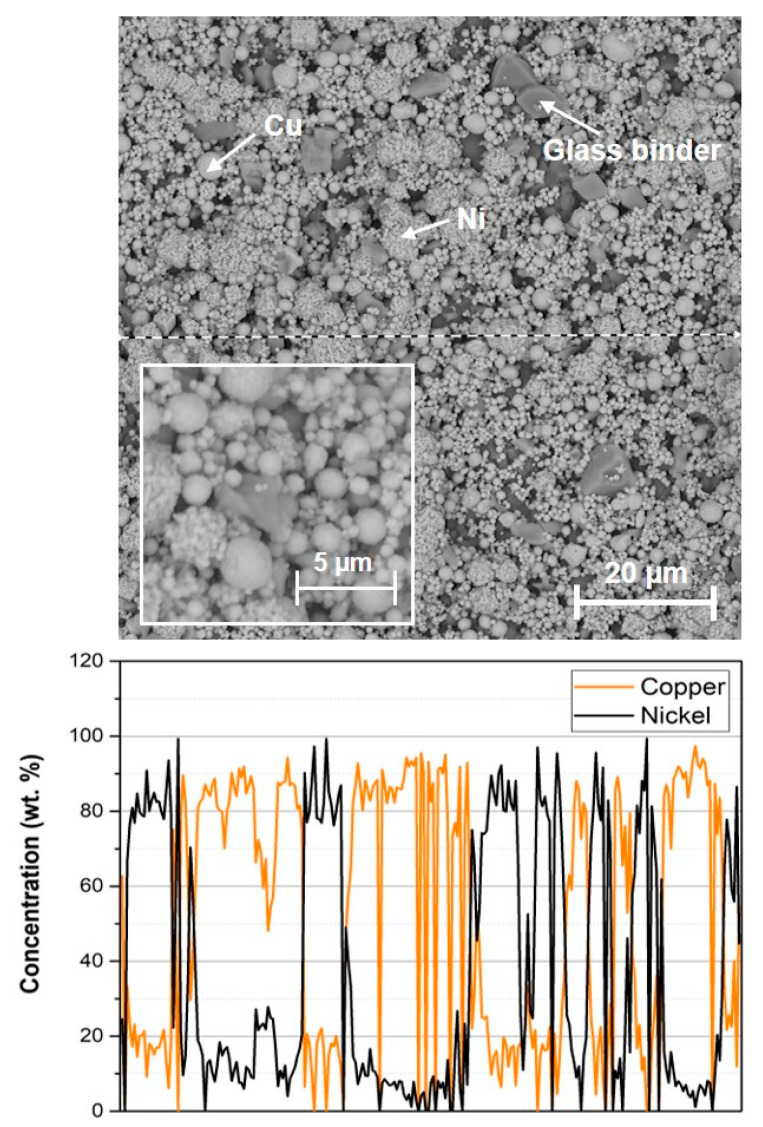
SEM image of dried copper-nickel thick film paste and EDS analysis of element distribution (dashed line represents the area where the EDS line scan was done).

SEM image of the dried copper-nickel film is shown in Figure 5. There are visible copper particles, conglomerates of nickel particles and glass binder particles. EDS analysis clearly shows areas with only copper or nickel corresponding to the distribution of copper and nickel particles. Copper and nickel particles are distributed uniformly and homogenously. The square box in Figure 5 represents the typical microstructure of the copper-nickel paste after drying at higher magnification while the rest of the figure shows the same paste at lower magnification, where it is easier to observe the homogeneity of the paste. Dark grey particles in Figure 5 represent a glass binder composed of 44.2 wt. % of oxygen, 14.7 wt. % of silicon, 11.8 wt. % of calcium, and 9.6 wt. % of aluminium.

**Figure 6:**
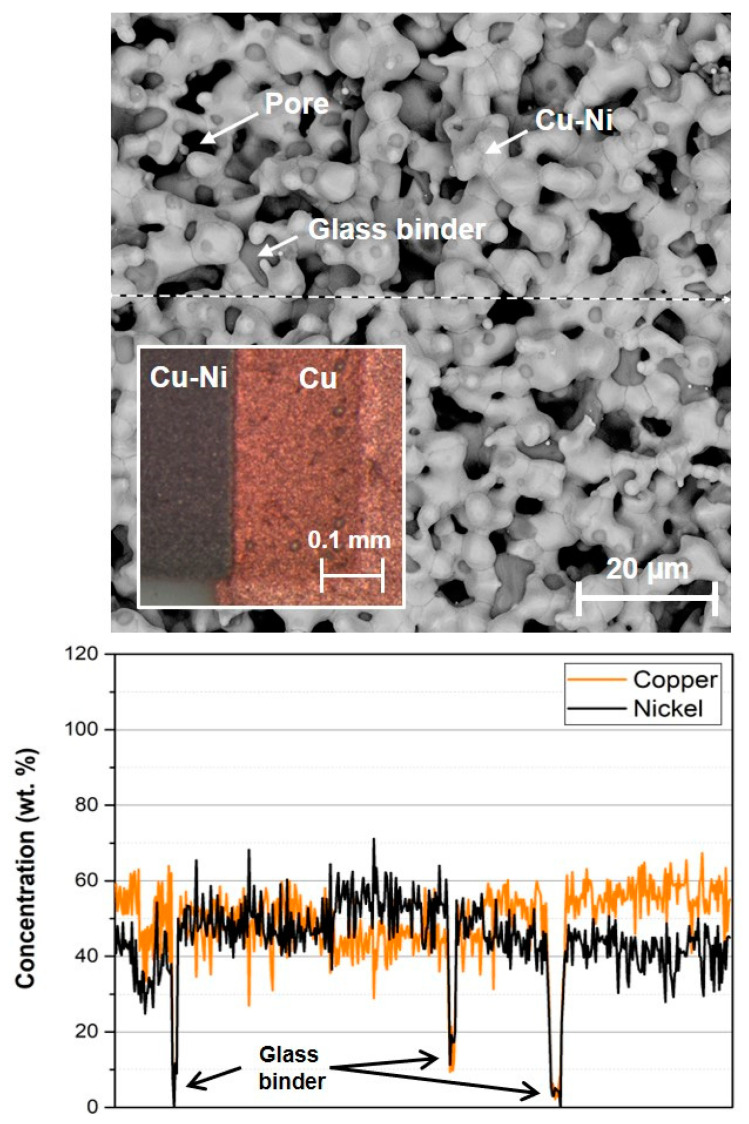
SEM image of fired copper-nickel thick film paste and EDS analysis of element distribution (dashed line represents the area where the EDS line scan was done).

The SEM image of the fired copper-nickel film is shown in Figure 6. It is visible that copper-nickel film is well sintered and copper and nickel particles are well diffused. EDS analysis confirmed that Cu and Ni particles diffused together and created Cu-Ni alloy with a ratio of approximately 55:45 wt. %. This fact is visible in the EDS analysis of element distribution in Figure 6 where is the homogenous distribution of copper-nickel alloy compared to EDS analysis in Figure 5 where are visible areas with only copper and nickel. The structure of the fired copper-nickel film is porous with 5–10 µm grain size. Fired copper-nickel film also contains the remains of glass binder on the surface. The glass binder, which is a part of the paste composition, melts during firing and the majority of it penetrates to interface between copper-nickel film and Al_2_O_3_ substrate where it ensures proper adhesion. Only a lower amount of glass binder remains in the copper-nickel film. These glass binder residues are also visible in EDS analysis of element distribution (areas with a low concentration of copper and nickel—Figure 6). The interface between copper (terminals) and copper-nickel films is visibly good without any cracks (Figure 6). The quality of this interface is very important for achieving the required properties (resistance and TCR) and the reliability of the final printed resistor.

**Figure 7:**
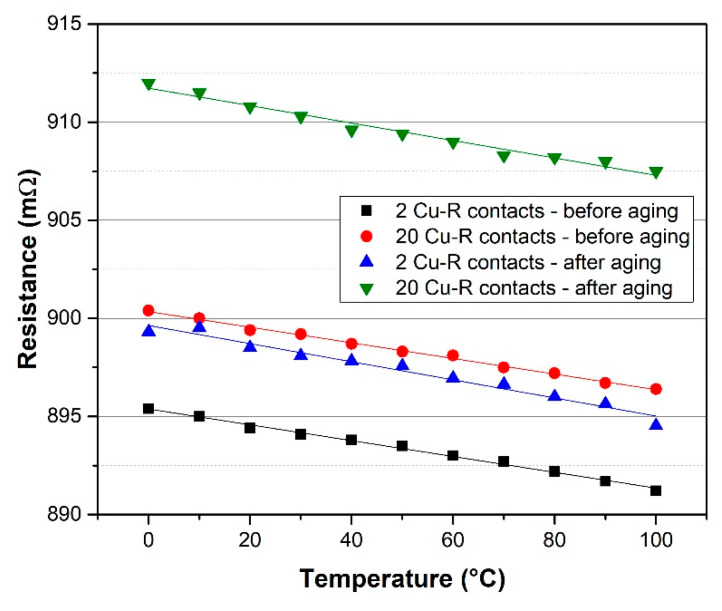
Temperature dependence of resistance of IRP specimens before and after accelerated aging.

The results of IRP specimen electrical measurement are listed in Table 1. Specimens with 2 Cu-R and 20 Cu-R had similar average nominal resistance values (894.0 mΩ in the case of 2 Cu-R contacts and 899.0 mΩ in the case of 20 Cu-R contacts). Nearly identical nominal resistance values of both types of specimens indicate that contact resistance formed at the interface of copper-nickel film and copper terminals was 0.5 mΩ per two Cu-R contacts. Contact resistance was calculated from the resistance difference of specimens with 2 Cu-R and 20 Cu-R contacts. This contact resistance was significantly lower as compared to resistors printed with standard resistive paste described in previous research [23] where the contact resistance was in the range of tens of Ohms. Low contact resistance is the basic precondition for the realization of low-ohmic resistors. The TCR of IRP specimens was ±50 × 10^−6^ K^−1^ for 2 Cu-R contacts and ±45 × 10^−6^ K^−1^ for 20 Cu-R contacts. It is obvious that an increasing number of Cu-R contacts has no significant effect on TCR value. The measurement also proved that TCR of printed copper-nickel films was close to TCR of bulk constantan alloy (±40 × 10^−6^ K^−1^). Resistance and TCR values of IRP specimens measured after accelerated aging by dry heat test (at 155 °C for 1000 h according to the standard IEC 60068-2-2) are also mentioned in Table 1. Both resistance and TCR values did not significantly change after accelerated aging, which meets the prerequisites for the long-term stability of copper-nickel printed resistors. The temperature dependence of resistance of realized IRP specimens before and after accelerated aging are shown in Figure 7. It is visible that this dependence is linear with a negative slope for all specimens.

**Figure 8:**
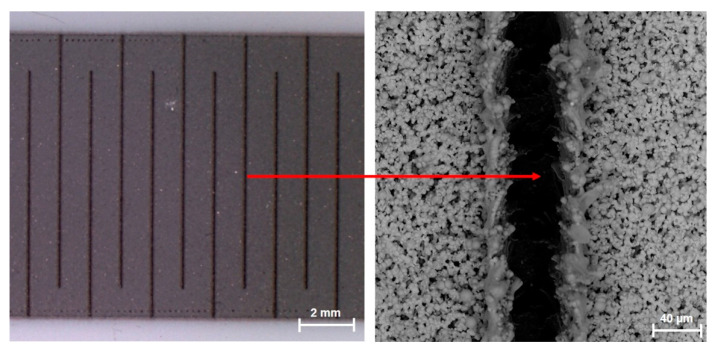
Copper-nickel resistor after laser trimming and SEM image of detail morphology of laser cut.

Not only are low absolute TCR values and the long-term stability of nominal resistance values of low-ohmic power resistors used mainly as a shunt or current sensing applications important, but accurate nominal resistance values are also required. It is very difficult to achieve accuracies better than ±10% with thick film technology using the screen-printing technique. Due to accurate nominal resistance value requirements, laser trimming of copper-nickel paste was verified using conventional YAG lasers. Laser trimming experiments proved that copper-nickel film allows adjustment of final nominal resistance value with the accuracy of ±0.5%. The laser trimming experiments were performed on laser machine L-TRIS LS-9600TD with laser source X30SC-106QA (1064 nm/40 W, Nd: YV04@1mJ: 10–60 ns @ 1–500 kHz) with the following settings: laser power: 50%, pulse frequency 7 kHz, laser speed: 4 mm/s. Figure 8 shows the example of a copper-nickel resistor after laser trimming and an SEM image of the detailed morphology of a laser cut. 

## 4. Conclusions

The new resistive copper-nickel thick film paste was formulated. It was verified that this paste is nitrogen-fireable and well-compatible with thick printed copper pastes. This combination allows the realization of power substrates with directly integrated low-ohmic resistors. 

The copper-nickel paste contains copper and nickel microparticles, glass binder particles (adhesion component), and a mixture of organic solvents designed for firing in a nitrogen atmosphere. Copper and nickel microparticles diffused together during firing and created homogenous copper-nickel alloy film (with a ratio of approximately 55:45) which shows a low absolute value of TCR ±45 × 10^−6^ K^−1^ and low sheet resistance value 45 mΩ/square. This is relatively significant progress compared to previous research where the following parameters were achieved: sheet resistance ~195 Ω/square and TCR ±120 × 10^−6^ K^−1^ in the case of standard resistive paste, and sheet resistance ~1 Ω/square and TCR ±100 × 10^−6^ K^−1^ in the case of copper-nickel nanoparticle ink deposited by Aerosol Jet. 

It was also verified that, between copper terminals and the copper-nickel resistive film, a stable metallurgical interface is created with low contact resistance (0.5 mΩ per one resistor). Low contact resistance is a basic precondition for the realization of low-ohmic printed resistors. These resistors can be predominantly used for shunt and current sensing applications on power electronic modules, where their direct integration to the substrate brings benefits of better heat dissipation from the whole area of the resistor to the substrate and subsequently to heat sink, lower parasitic inductance, significantly higher operating temperature, and better reliability due to a lack of soldering joints.

## Figures and Tables

**Table 1:** Results of copper-nickel IRP specimens measurement (average values from 5 specimens).

Specimen	Thickness of Cu-Ni Film (µm)	Resistance (mΩ)	Sheet Resistance (mΩ/square)	TCR (10^−6^ K^−1^)	Resistance after Aging (mΩ)	TCR after Aging (10^−6^ K^−1^)
2 Cu-R contacts	45	894.0	44.7	±50.0	898.5	±52.1
20 Cu-R contacts		899.0	45.0	±45.0	910.8	±49.6

## Data Availability

All data are described in the paper.
